# Changes in views on digital intraoral scanners among dental hygienists after training in digital impression taking

**DOI:** 10.1186/s12903-015-0140-5

**Published:** 2015-11-27

**Authors:** Hye-Ran Park, Ji-Man Park, Youn-Sic Chun, Kkot-Nim Lee, Minji Kim

**Affiliations:** Graduate School of Clinical Dentistry, Ewha Womans University, Seoul, Korea; Department of Prosthodontics and Dental Research Institute, Seoul National University Gwanak Dental Hospital, Seoul, Korea; Department of Orthodontics, Graduate School of Clinical Dentistry, Ewha Womans University, 1071, Anyangcheon-ro, Yangcheon-gu, Mokdong Hospital, Seoul, Korea

**Keywords:** Digital intraoral scanner, Perception of digital impression, Training of intraoral scanner

## Abstract

**Backgrounds:**

Despite the rapid development of digital dentistry, the use of digital intraoral scanners remains limited. The aim of this study was to evaluate the changes in views on intraoral scanners among dental hygienists after training.

**Methods:**

Thirty-four dental hygienists with >3 years of clinical experience participated and were divided into 2 groups : iTero and Trios groups. Participants of each group practiced the usage of both intraoral scanners, for total 12 times over 4 sessions, Questionnaires were given to participants at two different times; prior to and after the completion of the training sessions. The parameters of questionnaires included on difficulty of use, patient discomfort, awareness, preference, and clinical usefulness of intraoral scanners and comparison of two types of scanners.

**Results:**

Upon the completion of the training, both iTero and Trios groups gave positive feedback on anticipated accuracy, efficiency, and clinical usefulness. More participants of the iTero group responded that the level of difficulty of use and patient discomfort was greater than Trios. Both groups preferred Trios for its clinical usefulness.

**Conclusions:**

The perceptions of dental hygienists on usage of intraoral scanner and digital impression improved positively with the training. The participants favored Trios over iTero in terms of difficulty of use , patient comfort, and clinical usefulness. This study showed that appropriate training could change the views on the efficiency of intraoral scanners positively among dental hygienists.

**Electronic supplementary material:**

The online version of this article (doi:10.1186/s12903-015-0140-5) contains supplementary material, which is available to authorized users.

## Backgrounds

Computer-aided design/computer-aided manufacture (CAD/CAM) was first introduced in the field of dentistry in the 1980s; since then, the use of digital technology has been rapidly increased in dentistry, though having some arguments on its accuracy [1–4]. The conventional impression-taking method may pose patient discomfort and possibility of deformation which could be affected by the type of impression material [5–9], impression tray [10–12], and impression technique [13]. The digital impression technique could overcome these limitations by providing simple operating system [2, 14], accuracy, and improved patient comfort [15, 16]. Additional advantages include providing a preview of three-dimensional (3D) images while taking the impression. Some studies evaluated on accuracy of digital impression technique using intraoral scanners and reported that accuracy could be affected by materials or scanning strategies [2, 4]. Previous studies on the digital impression-taking using intraoral scanners have been limited to the accuracy and efficiency of intraoral scanners [17–19]. Some of previous studies compared the inconvenience and difficulty of digital impression method using between intraoral scanners compared to conventional impression methods among dental students in the university [15, 20, 21]. The aim of this study is to evaluate the changes in views on intraoral scanners among dental hygienists after training in digital impression.

## Methods

### Participants

Thirty-four dental hygienists with a clinical experience of at least 3 years who had no experience in using intraoral scanners participated in the study and they were divided into 2 groups ; an iTero (n = 17) and a Trios (n = 17) group using random allocation method. All participants were recruited voluntarily after the informative session about the study and written consent forms were obtained. This study was approved by the Institutional Review Board Committee of Ewha Womans University Medical College (Approval number: ECT14-02A-27).

### Intraoral scanners

In this study, 2 different types of 3D intraoral scanners, iTero® (Align Technology Inc. Santa Clara, California) and Trios® (3Shape dental systems, Copenhagen, Denmark), were used. The iTero is operated by the parallel confocal principle and acquires 3D data by over 100,000 red laser beams to the object and fusing the acquired images.The weight of the wand is 1,100 g. The Trios is operated by the confocal principle with the video-recording method based on the real-time rendering technique. The scanner head of Trios weighs 760 g. Both intraoral scanners were operated according to the manufacturer’s instructions.

### Study design and Workflow 

**Fig. 1:**
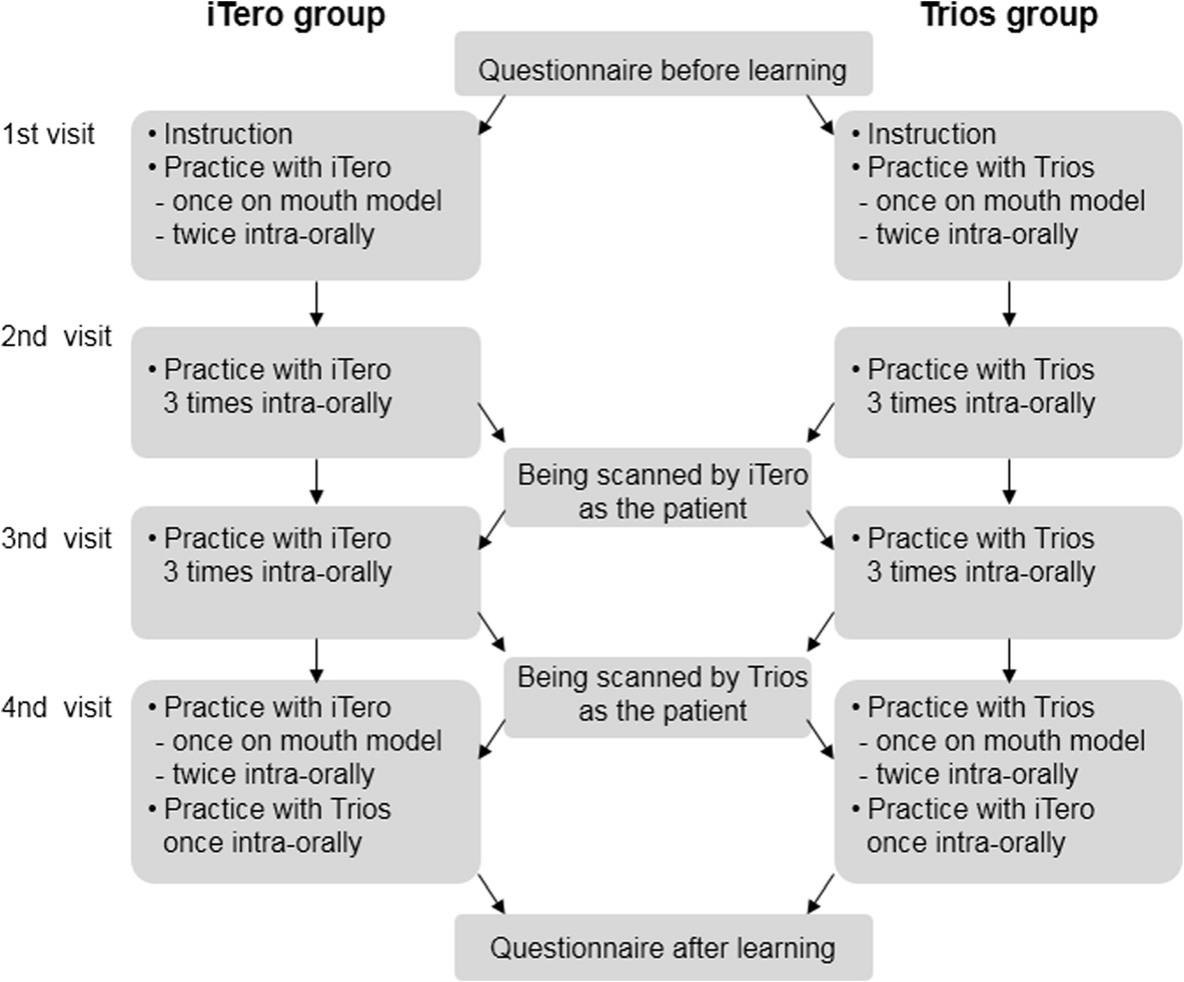
Procedure workflow

All participants not only performed as operators, but also underwent to have scanning experience as patients. The participants were fully trained with only one scanner according to assigned group, and the other scanner was used once for the comparing purpose between two different scanners. On the first day, each group of participants filled out the first questionnaire before the training was initiated. Then, the principles and operational concepts of iTero and Trios intraoral scanner devices were introduced. Practice session of intraoral scanner on models was followed by actual intraoral scanning activity. Digital impression from actual intraoral scan was obtained twice. On the second day, each group of participants practiced and took intraoral digital impression for three times. And all participants also underwent for iTero scanning experience as patients, which was operated by a qualified professional. On the third day, all participants again acquired actual intraoral images for 3 times. And they underwent Trios scanning experience as patients, which was operated by a qualified professional. On the last day, all participants acquired actual intraoral digital impression for twice and then practiced once on models again. Finally, each group of participants exchanged to the other scanner and acquired digital impression once intraorally. Upon the completion of the training sessions, all participants completed the second questionnaire.(Fig. 1)

### Questionnaire configuration 

Two questionnaires were administered during the study (Additional file 1). The first preliminary questionnaire was given initially before the training sessions and the second questionnaire was completed upon the completion of all the training sessions. The first questionnaire was administered before the training sessions and evaluated three main parameters: difficulties of using intraoral scanners with digital impression method compared to conventional impression-taking method, patient comfort, and degree of awareness about intraoral scanners. The parameter of awareness included anticipated accuracy, patient convenience, efficiency, clinical application, and interest in further use. The second questionnaire was administered upon the completion of all the training sessions and evaluated all the above parameters in addition to parameters of preference, clinical usefulness, and comparison of the two different types of scanners.

### Statistical analysis

All statistical analyses were performed using IBM Statics 19.0 (SPSS Inc., Chicago, USA) software. The paired t-test was used to test differences. The level of significance was set at 0.05.

## Results

### Views on difficulty of use and patient comfort before and after training

There was no significant difference in views on difficulties of using intraoral scanner devices for digital impression-taking method compared to that with conventional impression-taking methods before and after training in both the iTero and Trios groups (Table 1). A similar result was shown for parameter of patient comfort.

**Table 1:** Difficulty of use and patient discomfort while using intraoral scanners and rubber and alginate materials, before and after training (10-point Likert scale)

Variables	Conventional impression material	iTero group			Trios group		
		Mean (SD)		*p*-value	Mean (SD)		*p-*value
		Before	After		Before	After	
Level of difficulty	Rubber	4.65 (2.09)	5.65 (1.94)	.101	5.12 (1.80)	5.00 (2.26)	.847
	Alginate	6.18 (2.38)	7.29 (1.65)	.128	5.77 (2.46)	5.59 (2.62)	.779
Level of Patient discomfort	Rubber	4.29 (2.05)	4.12 (1.83)	.726	4.47 (2.63)	4.47 (2.79)	1.000
	Alginate	4.88 (2.29)	5.18 (2.33)	.663	4.24 (2.10)	4.47 (2.76)	.680

10-point Likert scale: 0 ~ 4 = low score (Digital impression taking was easier and more convenient) 5; neutral; 6 ~ 10 = high score (Digital impression taking was difficult and inconvenient)

**Fig. 2:**
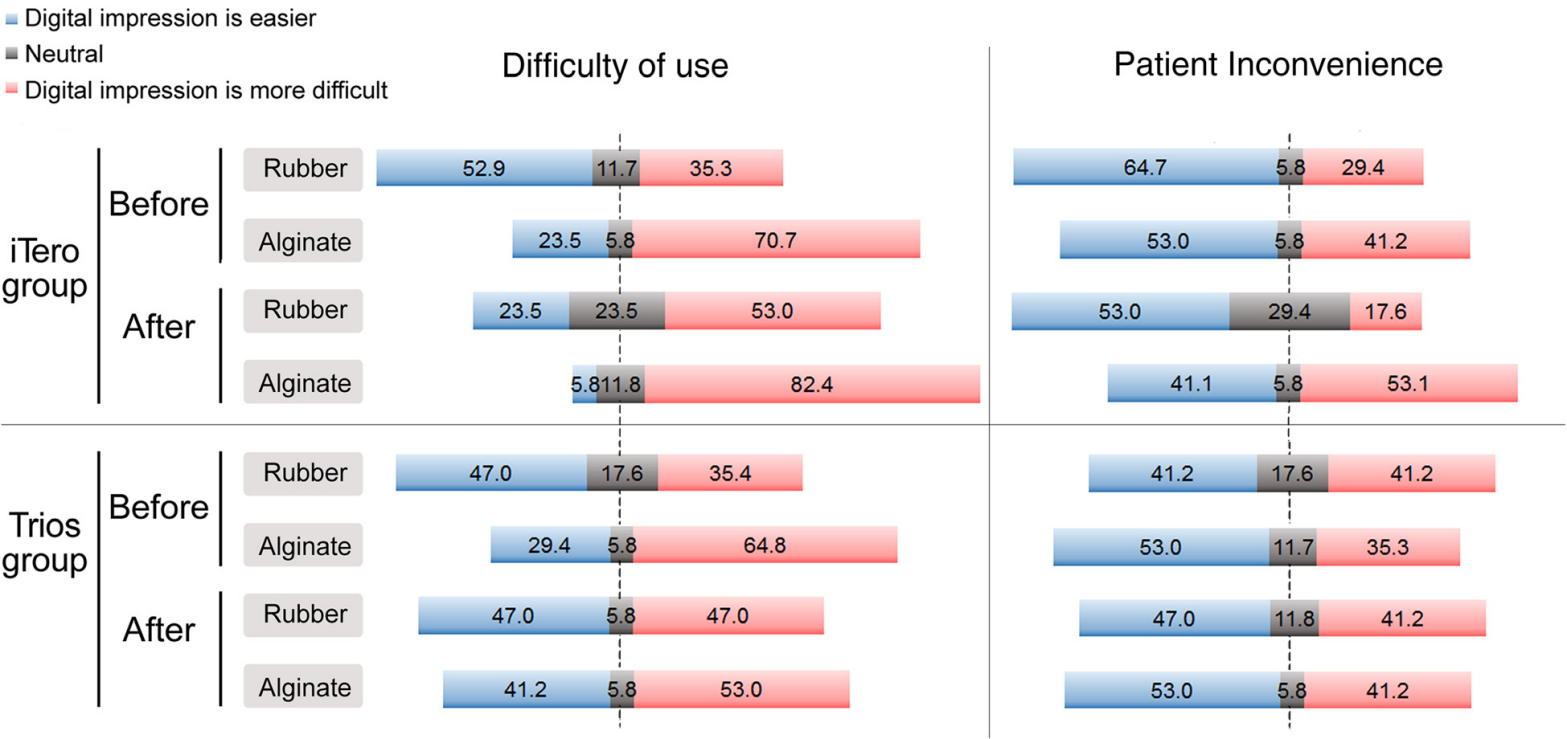
Difficulty of use and patient inconvenience between intraoral scanners and rubber and alginate materials before and after training (10-point Likert scale)

In the iTero group, prior to the training sessions, 53 % of participants responded that digital impression taking method may be easier than taking conventional impression using rubber materials. However, after the training sessions, only 24 % of participants in iTero group responded that digital impression taking method may be easier compared to conventional rubber impression taking method (Fig. 2). In the Trios group, the response rate for the same question was 47 %, prior to and after the training. Furthermore, when the difficulties of using digital impression taking method was compared to the conventional impression taking method using alginate material the participants responded digital impression to be more difficult as 71 % and 82 % before and after training, respectively, in the iTero group, and 65 % and 53 % before and after training, respectively, in the Trios group.

To evaluate parameter of patient comfort, all the participants underwent digital impression scanning experience using iTero and Trios. Before the training, 65 % of participants in the iTero group and 41 % of participants in the Trios group responded that digital impression taking may be more comfortable to patients than impression taking using rubber materials; these rates decreased to 53 % in the iTero group and increased to 47 % in the Trios group. In comparison with patient comfort during impression taking using alginate, subjects who answered that digital impression taking was more convenient decreased from 53 % to 41 % in the iTero group and remained the same in the Trios group (53 %).

The number of subjects who responded that digital impression taking was easier than conventional impression taking as an operator was greater for rubber impression materials than for alginate. This was due to difficulty, and higher level of expected precision in the use of rubber materials. The number of responses that digital impression taking was more difficult generally increased after training in the iTero group and decreased in the Trios group. With regard to patient comfort, the number of responses that digital impression taking was more convenient was almost similar before and after training in both groups.

### Awareness about digital impression taking before and after training

As shown in Table 2, scores for the anticipated accuracy of digital impression taking method increased after the training in the iTero group. With regard to clinical usefulness, after the training sessions, more participants in the iTero group answered that digital scanners could be helpful tools in dental clinics. However, there were no significant differences for other parameters before and after training in the iTero group. In the Trios group, there were no significant differences for any parameters.

**Table 2:** Awareness about digital impression taking before and after training (7-point Likert scale)

Parameters	Variables (Scores for agreement regarding digital impression taking)	iTero			Trios		
		Mean (SD)		*p*-value	Mean (SD)		*p*-value
		Before	After		Before	After	
Accuracy	More accurate than rubber impression taking	4.29 (1.45)	4.82 (1.24)	0.024*	5.06 (1.09)	5.59 (1.23)	0.132
	More accurate than alginate impression taking	4.77 (1.60)	5.35 (1.62)	0.096	5.47 (1.13)	6.06 (1.09)	0.086
Convenience	More efficient management of impression model	5.88 (1.69)	6.29 (1.49)	0.130	6.41 (0.80)	6.59 (0.62)	0.484
Efficiency	Possibility of saving time compared to rubber impressions	5.24 (1.60)	4.88 (1.57)	0.455	4.65 (1.54)	4.82 (1.63)	0.661
	Possibility of saving time compared to alginate impressions	4.29 (2.14)	4.06 (2.05)	0.702	4.29 (1.72)	4.06 (1.78)	0.632
	Influence on simplification of the entire treatment process	5.24 (1.03)	5.06 (1.56)	0.627	5.18 (1.01)	5.47 (1.06)	0.311
	Usefulness in attracting patient’s attention.	5.82 (1.43)	6.12 (0.78)	0.385	5.47 (1.18)	6.00 (0.94)	0.083
Clinical usefulness	Influence on increasing patient’s trust.	5.35 (1.58)	5.88 (0.78)	0.120	5.59 (1.06)	5.77 (0.90)	0.616
	Influence on promoting the dental clinic.	5.35 (1.50)	5.88 (0.99)	0.034*	5.59 (1.06)	6.00 (0.87)	0.069
Skill acquisition	Ease of training in a short time	4.47 (1.28)	4.06 (1.30)	0.370	4.65 (1.46)	4.65 (1.73)	1.000
	Effect of proficiency in using digital impression techniques on the accuracy	6.24 (0.83)	6.59 (0.71)	0.251	6.18 (0.88)	6.18 (0.72)	1.000
Superior ability in taking digital impressions compared to other colleagues		5.18 (1.33)	5.41 (1.27)	0.299	5.65 (1.00)	5.71 (0.85)	0.817
Usefulness of digital impression in the clinical environment		4.82 (1.28)	5.18 (1.29)	0.370	5.47 (0.80)	5.53 (1.23)	0.848
Positive interest in taking digital impressions		5.59 (1.06)	5.29 (0.92)	0.206	5.65 (0.79)	5.71 (1.11)	0.848

7-point Likert scale: 1 = very strongly disagree, 2 = strongly disagree, 3 = disagree, 4 = neutral, 5 = agree, 6 = strongly agree, 7 = very strongly agree, *: *p* < 0.05

**Fig. 3:**
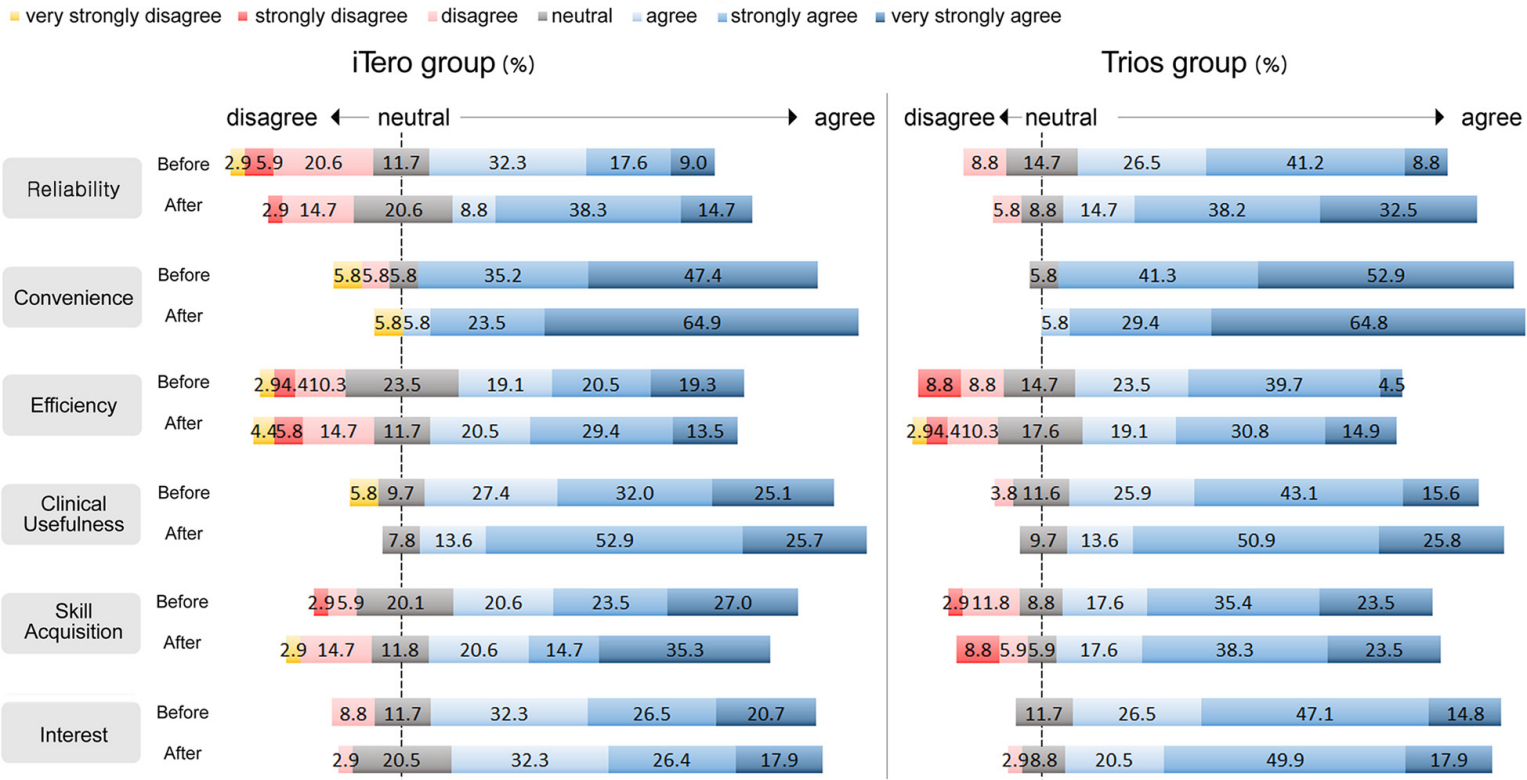
Awareness about digital impression taking before and after training (7-point Likert scale)

The participants from both groups generally showed positive responses for all parameters of digital impression taking method (Fig. 3).

### Preference for digital impression taking

**Fig. 4:**
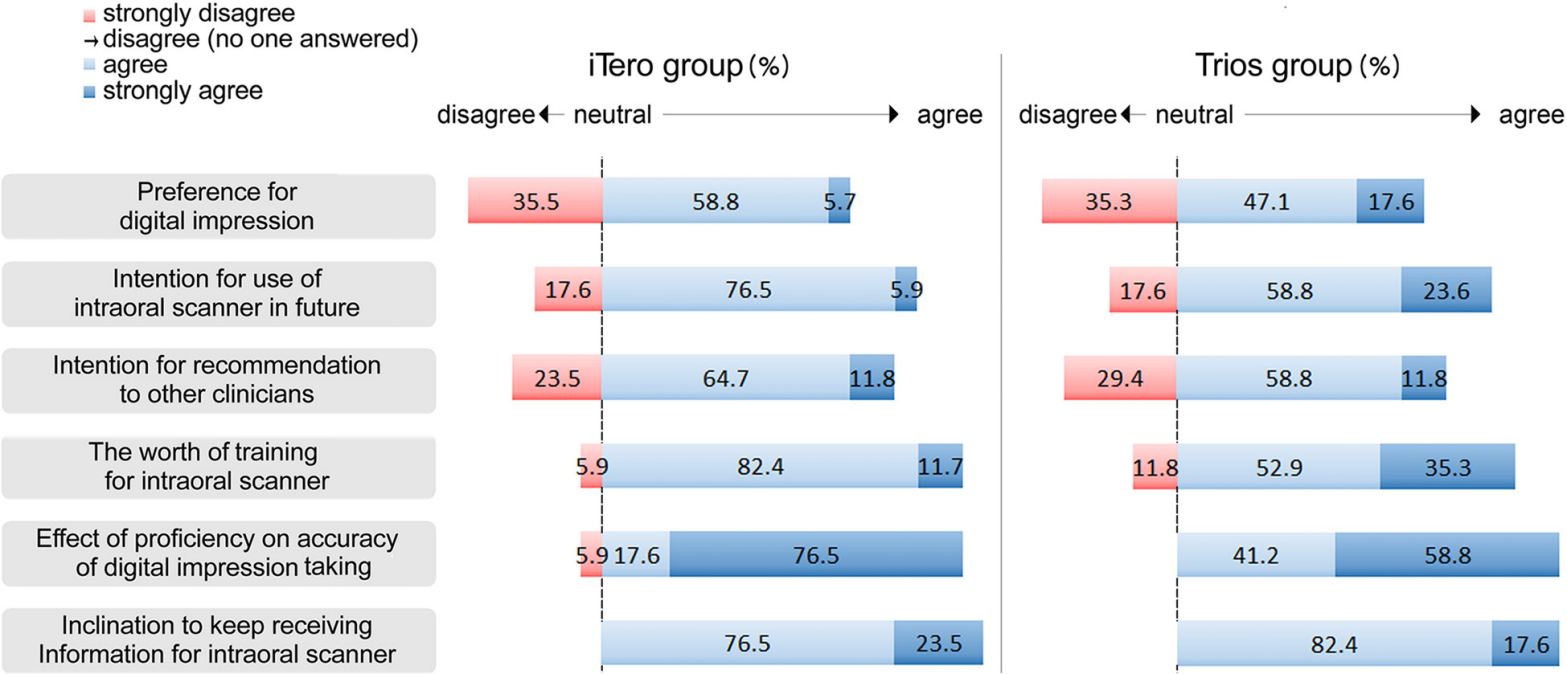
Preference for digital impression taking after training (4-point Likert scale). No subject responded “disagree”

As shown in Fig. 4, all participants from both groups gave a positive overall feedbacks for digital impression taking method. The results showed that 82.4 % participants of both iTero and Trios groups showed willingness to use the intraoral scanner in the future. Participants agreed that the training for the intraoral scanner is useful; 94.1 % participants in the iTero group and 88.2 % participants in the Trios group. All subjects were interested in receiving information about intraoral scanners.

### Views on clinical usefulness in the iTero and Trios groups

In terms of the subjective weight of the scanner head between two intraoral scanners, in the iTero group, the degree of agreement to the fact that the scanner head was light was significantly higher for Trios (2.65 ± 0.86) than for iTero (1.65 ± 0.78) (Table 3). Similar results were shown in Trios group. In the iTero group, the response that handling the scanner was convenient showed a significantly different degree of agreement for iTero (2.52 ± 0.87) and Trios (3.17 ± 0.52). With regard to the ease of software and hardware operation in the Trios group, more respondents agreed that Trios was easy to operate.

**Table 3:** Views on the clinical usefulness of iTero and Trios after training (4-point Likert scale)

Variables	iTero group			Trios group		
	iTero	Trios	*p-*value	iTero	Trios	*p*-value
	Mean (SD)			Mean (SD)		
Light weight head	1.65 (0.78)	2.65 (0.86)	.000*	1.89 (0.60)	2.30 (0.58)	.014*
Small-sized head	2.12 (0.69)	2.42 (0.87)	.096	2.00 (0.70)	2.18 (0.63)	.332
Easy to operate software	2.89 (0.85)	2.95 (0.82)	.750	2.24 (0.75)	3.00 (0.50)	.008*
Easy to operate hardware	2.47 (0.71)	2.82 (0.63)	.188	2.29 (0.46)	2.70 (0.46)	.004*
Good grip	2.05 (0.65)	2.76 (0.66)	.009*	2.35 (0.78)	2.23 (0.66)	.651
Quick training	2.23 (0.56)	2.70 (0.77)	.056	2.41 (0.50)	2.58 (0.50)	.269
Comfortable handling	2.52 (0.87)	3.17 (0.52)	.007*	2.58 (0.71)	2.70 (0.77)	.579

4-point Likert scale: 1 = strongly disagree, 2 = disagree, 3 = agree, 4 = strongly agree, *: *p* < 0.05

These results suggested that positive responses for clinical usefulness were greater with Trios than for iTero intraoral scanner.

### Views on difficulty of use and patient comfort in the iTero and Trios groups

As shown as Table 4, in the iTero group, the level of difficulty of use was rated higher for iTero (5.47 ± 2.37) than for Trios (4.23 ± 1.92), as was the level of patient comfort (4.11 ± 2.08, 3.11 ± 1.56, respectively). However, in the Trios group, the levels of difficulty and discomfort showed no significant difference between Trios and iTero.

**Table 4:** Views on difficulty of use and patient discomfort while using iTero and Trios (10-point Likert scale)

	iTero group			Trios group		
Variables	iTero	Trios	*p*-value	iTero	Trios	*p*-value
	Mean (SD)			Mean (SD)		
Level of difficulty	5.47 (2.37)	4.23 (1.92)	.034*	5.70 (2.08)	4.52 (2.26)	.061
Level of patient discomfort	4.11 (2.08)	3.11 (1.56)	.007*	4.70 (2.08)	4.11 (2.17)	.276

10-point Likert scale: 0 ~ 4; low score, easy and more convenient, 5; neutral, 6 ~ 10; high score, difficult and inconvenient, *: *p* < 0.05

## Discussion

The clinical application of digital impression method has been increased continuously due to some advantages; possibility of immediate fabrication of intraoral models [22], and no requirement of impression trays and materials [14, 16]. Previous study by Lee et al. [21] evaluated the efficiency, difficulty and participants’ preference of a digital impression compared with a conventional impression methods. The result of the study reported that 40 % participants of clinicians group agreed to the efficiency of digital impression taking while 53 % of disagreed. And 33 % of clinicians preferred digital impression taking, 37 % favored conventional impression taking, and 30 % had no preference. In addition, participants of dental students group perceived that digital impression was easier than conventional impression whereas the clinicians perceived that there was no difference between digital and conventional impression-taking methods. Yuzbasioglu et al. [15] reported that 100 % of participants preferred digital impression-taking method using CEREC compared to conventional impression-taking methods in the parameters of following: patient comfort, sensitivity, gag reflex and user friendliness.

In the present study, although the participants practiced using the intraoral scanners only 12 times, the difficulty of use was generally similar between intraoral scanners and rubber or alginate impression materials. However, a greater number of participants responded that conventional alginate impression taking was easier than digital impression taking in both the iTero and Trios groups before and after training. This could have resulted from less time required for training and participants’ familiarity with alginate impression material and technique. When digital impression-taking method was compared to conventional impression taking method with rubber materials, participants of both itero and Trios groups responded that intraoral scanners were more convenient. With regard to conventional impression taking with alginate material, participants in Trios group generally agreed that intraoral scanners were more convenient than alginate before and after training. These results were consistent with results of previous study [15]. Interestingly, the proportion of patients in the iTero group who answered that alginate impression taking was more comfortable than digital impression taking decreased from 64.8 % to 53 %.

This study showed that 58.9 % participants agreed to the efficiency of digital impression taking before training, and this percentage increased to 63.4 % after training. Furthermore, the participants gave positive feedbacks for anticipated accuracy, patient comfort, clinical usefulness, ease of skill acquisition, and interest in further use. In this study, difficulty of use and patient discomfort were lower with Trios than with iTero. Moreover, as shown in Table 3, many participants responded that the head of the iTero intraoral scanner was heavier compared to Trios. However, the software of Trios scanner system was easier to operate than iTero system. The difference in scanner weight between iTero and Trios was 340 g (iTero, 1,100 g; Trios, 760 g). In this study, the rate of positive response to questions investigating preference for digital impression taking was significant in the both iTero and Trios groups, suggesting the possibility of rapid digitalization in the field of dentistry. This study had a small sample size and a short duration for the training session, therefore, further studies should be conducted with larger sample size and prolonged period of training

## Conclusions

This study evaluated progressive change in perceptions of digital intraoral scanners with short period of training in digital impression method among dental hygienists. The parameters of evaluation included difficulty of use, patient comfort, preference, and clinical usefulness.

Within the limitations of this study, training in the use of intraoral scanners has changed the views of dental hygienists positively. And the result of this study indicated that participants generally preferred Trios intraoral scanner over iTero as a operator. The usefulness of intraoral scanner could be a successful alternative to conventional impression-taking with proper training and increased clinical experiences in digital impression method.

## Additional file

Additional file 1:
**Questionnaire.** (PDF 300 kb)
