# Supplementary material for: Changes in views on digital intraoral scanners among dental hygienists after training in digital impression taking
Source: BMC Oral Health. 2015 Nov 27;15:151. doi: 10.1186/s12903-015-0140-5 (PMC4662823; doi:10.1186/s12903-015-0140-5)
Supplement: Additional file 1: — Questionnaire. (PDF 300 kb) [file 12903_2015_140_MOESM1_ESM.pdf]

# Questionnaire

**"Digital impression taking" is a method performed using the portable medical device "Digital scanner (Intraoral scanner)", which is capable of three-dimensional scanning of dental arches and generating a digital imprint.**

## PART I (Before training)

Please select the option that best describes your **views** about digital impression taking (select only one option "V")

### 1. The expected difficulty of digital impression taking as a clinician

(1) Compared to impression taking using **rubber**, digital impression taking seems ______.

| Easy | | | | | Same | | | | | Difficult |
|---|---|---|---|---|---|---|---|---|---|---|
| 0 | 1 | 2 | 3 | 4 | 5 | 6 | 7 | 8 | 9 | 10 |

(2) Compared to impression taking using **alginate**, digital impression taking seems ______.

| Easy | | | | | Same | | | | | Difficult |
|---|---|---|---|---|---|---|---|---|---|---|
| 0 | 1 | 2 | 3 | 4 | 5 | 6 | 7 | 8 | 9 | 10 |

### 2. The expected inconvenience caused to patients during digital impression taking

(1) Compared to impression taking using **rubber**, digital impression taking seems ______.

| Convenient | | | | | Same | | | | | Inconvenient |
|---|---|---|---|---|---|---|---|---|---|---|
| 0 | 1 | 2 | 3 | 4 | 5 | 6 | 7 | 8 | 9 | 10 |

(2) Compared to impression taking using **alginate**, digital impression taking seems ______.

| Convenient | | | | | Same | | | | | Inconvenient |
|---|---|---|---|---|---|---|---|---|---|---|
| 0 | 1 | 2 | 3 | 4 | 5 | 6 | 7 | 8 | 9 | 10 |

Please select the option that best describes your **views** about digital impression taking (select only one option "V")

3. In taking an impression using a digital scanner in the clinical environment,

| | Strongly disagree | Moderately disagree | Slightly disagree | Neutral | Slightly agree | Moderately agree | Strongly agree |
|---|---|---|---|---|---|---|---|
| (1) I think it is more reliable than impression taking using **rubber**. | 1 | 2 | 3 | 4 | 5 | 6 | 7 |
| (2) I think it is more reliable than impression taking using **alginate**. | 1 | 2 | 3 | 4 | 5 | 6 | 7 |
| (3) I think model management will get more convenient. | 1 | 2 | 3 | 4 | 5 | 6 | 7 |
| (4) I think it can save time in comparison with impression taking using **rubber**. | 1 | 2 | 3 | 4 | 5 | 6 | 7 |
| (5) I think it can save time in comparison with taking using **alginate**. | 1 | 2 | 3 | 4 | 5 | 6 | 7 |
| (6) I think the entire treatment process will become simpler. | 1 | 2 | 3 | 4 | 5 | 6 | 7 |
| (7) I think it will be useful for attracting the patient's attention. | 1 | 2 | 3 | 4 | 5 | 6 | 7 |
| (8) I think it can gain the patient's trust. | 1 | 2 | 3 | 4 | 5 | 6 | 7 |
| (9) I think it will be helpful for promoting the dental clinic. | 1 | 2 | 3 | 4 | 5 | 6 | 7 |
| (10) I think it can be learned and mastered in a short time. | 1 | 2 | 3 | 4 | 5 | 6 | 7 |
| (11) I think the degree of proficiency will affect accuracy. | 1 | 2 | 3 | 4 | 5 | 6 | 7 |

| | Strongly disagree | Moderately disagree | Slightly disagree | Neutral | Slightly agree | Moderately agree | Strongly agree |
|---|---|---|---|---|---|---|---|
| 4. I think that if I take a digital impression, I will get ahead of another colleague who does not use it. | 1 | 2 | 3 | 4 | 5 | 6 | 7 |
| 5. I think taking an impression using a digital scanner is generally useful in the clinical environment. | 1 | 2 | 3 | 4 | 5 | 6 | 7 |
| 6. Overall, I prefer taking impressions using a digital scanner in the clinical environment. | 1 | 2 | 3 | 4 | 5 | 6 | 7 |

## PART II (After training)

Please select the option that best describes your **views** about digital impression taking (select only one option “V”)

### 1. The difficulty of digital impression taking as a clinician

(1) Compared to impression taking using **rubber**, digital impression taking was ______.

| Easy | | | | | Same | | | | | Difficult |
|---|---|---|---|---|---|---|---|---|---|---|
| 0 | 1 | 2 | 3 | 4 | 5 | 6 | 7 | 8 | 9 | 10 |

(2) Compared to impression taking using **alginate**, digital impression taking was ______.

| Easy | | | | | Same | | | | | Difficult |
|---|---|---|---|---|---|---|---|---|---|---|
| 0 | 1 | 2 | 3 | 4 | 5 | 6 | 7 | 8 | 9 | 10 |

---

### 2. The inconvenience caused to patients during digital impression taking

(1) Compared to impression taking using **rubber**, digital impression taking was ______.

| Convenient | | | | | Same | | | | | Inconvenient |
|---|---|---|---|---|---|---|---|---|---|---|
| 0 | 1 | 2 | 3 | 4 | 5 | 6 | 7 | 8 | 9 | 10 |

(2) Compared to impression taking using **alginate**, digital impression taking was ______.

| Convenient | | | | | Same | | | | | Inconvenient |
|---|---|---|---|---|---|---|---|---|---|---|
| 0 | 1 | 2 | 3 | 4 | 5 | 6 | 7 | 8 | 9 | 10 |

Please select the option that best describes your **views** about digital impression taking (select only one option “V”)

3. In taking an impression using a digital scanner in the clinical environment,

| | Strongly disagree | Moderately disagree | Slightly disagree | Neutral | Slightly agree | Moderately agree | Strongly agree |
|---|---|---|---|---|---|---|---|
| (1) I think it is more reliable than impression taking using **rubber**. | 1 | 2 | 3 | 4 | 5 | 6 | 7 |
| (2) I think it is more reliable than impression taking using **alginate**. | 1 | 2 | 3 | 4 | 5 | 6 | 7 |
| (3) I think model management will get more convenient. | 1 | 2 | 3 | 4 | 5 | 6 | 7 |
| (4) I think it can save time in comparison with impression taking using **rubber**. | 1 | 2 | 3 | 4 | 5 | 6 | 7 |
| (5) I think it can save time in comparison with taking using **alginate**. | 1 | 2 | 3 | 4 | 5 | 6 | 7 |
| (6) I think the entire treatment process will become simpler. | 1 | 2 | 3 | 4 | 5 | 6 | 7 |
| (7) I think it will be useful for attracting the patient’s attention. | 1 | 2 | 3 | 4 | 5 | 6 | 7 |
| (8) I think it can gain the patient’s trust. | 1 | 2 | 3 | 4 | 5 | 6 | 7 |
| (9) I think it will be helpful for promoting the dental clinic. | 1 | 2 | 3 | 4 | 5 | 6 | 7 |
| (10) I think it can be learned and mastered in a short time. | 1 | 2 | 3 | 4 | 5 | 6 | 7 |
| (11) I think the degree of proficiency will affect accuracy. | 1 | 2 | 3 | 4 | 5 | 6 | 7 |

| | Strongly disagree | Moderately disagree | Slightly disagree | Neutral | Slightly agree | Moderately agree | Strongly agree |
|---|---|---|---|---|---|---|---|
| 4. I think that if I take a digital impression, I will get ahead of another colleague who does not use it. | 1 | 2 | 3 | 4 | 5 | 6 | 7 |
| 5. I think taking an impression using a digital scanner is generally useful in the clinical environment. | 1 | 2 | 3 | 4 | 5 | 6 | 7 |
| 6. Overall, I prefer taking impressions using a digital scanner in the clinical environment. | 1 | 2 | 3 | 4 | 5 | 6 | 7 |

# PART III (After training)

Please select the option that best describes your **preferences** regarding digital impression taking (select only one option “V”)

1. In comparison with the conventional method of taking an impression, do you prefer the method of taking impressions using a digital intraoral scanner?

   ① Strongly disagree (Not at all) ② Disagree (No) ③ Agree (Yes) ④ Strongly agree (Completely)

2. Are you willing to continue using a digital intraoral scanner?

   ① Strongly disagree (Not at all) ② Disagree (No) ③ Agree (Yes) ④ Strongly agree (Completely)

3. Are you willing to recommend a digital intraoral scanner to people around you?

   ① Strongly disagree (Not at all) ② Disagree (No) ③ Agree (Yes) ④ Strongly agree (Completely)

4. Do you think it is worth investing time in learning how to use a digital intraoral scanner?

   ① Strongly disagree (Not at all) ② Disagree (No) ③ Agree (Yes) ④ Strongly agree (Completely)

5. Do you think that there is a difference in accuracy between digital impression taking by a skilled person and a beginner?

   ① Strongly disagree (Not at all) ② Disagree (No) ③ Agree (Yes) ④ Strongly agree (Completely)

6. Would you like to keep receiving information about digital intraoral scanners?

   ① Strongly disagree (Not at all) ② Disagree (No) ③ Agree (Yes) ④ Strongly agree (Completely)

# PART IV (After training)

You have received training to use the digital intraoral scanners iTero and Trios.

Please select the option that best describes your opinion about their **clinical usefulness** (select only one option "V")

## About iTero

1. I think the head of the iTero unit is light weight.
   ① Strongly disagree (Not at all) ② Disagree (No) ③ Agree (Yes) ④ Strongly agree (Completely)

2. I think the head size of iTero is small.
   ① Strongly disagree (Not at all) ② Disagree (No) ③ Agree (Yes) ④ Strongly agree (Completely)

3. I think the software management of iTero is easy.
   ① Strongly disagree (Not at all) ② Disagree (No) ③ Agree (Yes) ④ Strongly agree (Completely)

4. I think it is possible to quickly master the use of the iTero scanner.
   ① Strongly disagree (Not at all) ② Disagree (No) ③ Agree (Yes) ④ Strongly agree (Completely)

5. I think the iTero unit provides a good grip.
   ① Strongly disagree (Not at all) ② Disagree (No) ③ Agree (Yes) ④ Strongly agree (Completely)

6. I think handing the iTero unit is convenient.
   ① Strongly disagree (Not at all) ② Disagree (No) ③ Agree (Yes) ④ Strongly agree (Completely)

## About Trios

1. I think the head of the Trios is light weight.
   ① Strongly disagree (Not at all) ② Disagree (No) ③ Agree (Yes) ④ Strongly agree (Completely)

2. I think the head size of Trios is small.
   ① Strongly disagree (Not at all) ② Disagree (No) ③ Agree (Yes) ④ Strongly agree (Completely)

3. I think the software management of Trios is easy.
   ① Strongly disagree (Not at all) ② Disagree (No) ③ Agree (Yes) ④ Strongly agree (Completely)

4. I think it is possible to quickly master the use of the Trios scanner.
   ① Strongly disagree (Not at all) ② Disagree (No) ③ Agree (Yes) ④ Strongly agree (Completely)

5. I think the Trios unit provides a good grip.
   ① Strongly disagree (Not at all) ② Disagree (No) ③ Agree (Yes) ④ Strongly agree (Completely)

6. I think handling the Trios unit is convenient.
   ① Strongly disagree (Not at all) ② Disagree (No) ③ Agree (Yes) ④ Strongly agree (Completely)

# PART V (After training)

You have received training to use the digital intraoral scanners iTero and Trios.

Please select the option that best describes your views about the two intraoral scanners (select only one option “V”)

1. The difficulty of digital impression taking as a clinician

(1) When scanning with iTero

Easy ← Same → Difficult

0 1 2 3 4 5 6 7 8 9 10

(2) When scanning with Trios

Easy ← Same → Difficult

0 1 2 3 4 5 6 7 8 9 10

---

2. The inconvenience caused to patients during digital impression taking

(1) When undergoing scanning with iTero

Convenient ← Same → Inconvenient

0 1 2 3 4 5 6 7 8 9 10

(2) When undergoing scanning with Trios

Convenient ← Same → Inconvenient

0 1 2 3 4 5 6 7 8 9 10
